# Timeliness of cancer care in a regional Victorian health service: A comparison of high‐volume (Lung) and low‐volume (oesophagogastric) tumour streams

**DOI:** 10.1002/cnr2.1301

**Published:** 2020-10-07

**Authors:** Mwila Kabwe, Amanda Robinson, Yachna Shethia, Carol Parker, Robert Blum, Ilana Solo, Michael Leach

**Affiliations:** ^1^ Loddon Mallee Integrated Cancer Service Bendigo Health Bendigo Victoria Australia; ^2^ Department of Pharmacy and Biomedical Sciences La Trobe Institute for Molecular Science, La Trobe University Bendigo Victoria Australia; ^3^ Rural Health Monash University Bendigo Victoria Australia

**Keywords:** lung cancer, oesophagogastric cancer, optimal care pathway, timeliness of care

## Abstract

**Background:**

Timeliness of cancer care is vital for improved survival and quality of life of patients. Service and care centralisation at larger‐volume centres has been associated with improved outcomes. However, there is a lack of systematic data on the impact of tumour stream volume on timeliness of care.

**Aims:**

To investigate and compare timeliness of care for lung cancer, a high‐volume (more commonly diagnosed) tumour stream, and oesophagogastric (OG) cancer, a low‐volume (less commonly diagnosed) tumour stream, at a regional health service in Victoria, Australia.

**Methods:**

A retrospective cohort study comprising random samples of 75 people newly diagnosed with lung cancer (International Classification of Diseases and Related Health Problems‐10 [ICD‐10] diagnosis codes C34 in the Victorian Cancer Registry [VCR]) and 50 people newly diagnosed with OG cancer (ICD‐10 diagnosis codes C15 or C16 in VCR) at one regional Victorian health service between 2016 and 2017. Binary logistic regression was used to calculate odds ratios (ORs) and 95% confidence intervals (CIs) for associations between patient factors and suboptimal timeliness of care.

**Results:**

In comparison to OG cancer patients, lung cancer patients had reduced odds of suboptimal timeliness of care in reference to times outside OCP for referral to diagnosis (OR [95% CI] = 0.34 [0.14 to 0.83]) but increased odds of suboptimal timeliness for diagnosis to treatment (OR [95% CI] = 2.48 [1.01 to 6.09]).

**Conclusion:**

In the low‐volume OG cancer stream, patients had longer wait times from referral to an MDM, where treatment decisions occur, but shorter time to commencement of first treatment. Conversely in the high‐volume lung cancer group, there was delayed initiation of first treatment following presentation at MDM. There is need to explore ways to fast‐track MDM presentation and commencement of therapy among people diagnosed with low‐volume and high‐volume cancers, respectively.

## 1 INTRODUCTION

Cancer remains the leading cause of death worldwide.^1^ Lung cancer is the leading cause of cancer‐related mortality globally, accounting for 19% of all cancer‐related deaths.^2^ The incidence of lung cancer is second only to prostate cancer in men and breast cancer in women,^3^ making it a high‐volume tumour stream. Oesophagogastric (OG) cancer, on the other hand, is a low‐volume (less common) cancer.^4^ In Australia, the 5‐year survival rate in the last 10 years has been shown to be 17.4% in lung cancer and 22.0% in OG cancer.^5^

Most factors associated with early deaths due to cancer are pathological, such as stage at diagnosis and site of metastasis.^6^, ^7^, ^8^ Hence, factors associated with early cancer‐related death may be different for each tumour stream. Overall, however, one of the fundamental determinants of disease outcome and patient experience is timeliness of care.^9^ Diagnosing cancer early to help ensure better survival has, for a long time, been difficult to achieve.^10^

In Australia, Cancer Council Victoria has produced nationally‐endorsed optimal care pathways (OCPs) for people with cancer.^11^ These pathways apply to particular cancer streams and are designed to reduce unwarranted variation in care, including timeliness of care.^11^ The OCPs include optimal timeframe recommendations for times between key aspects of care, such as referral from the general practitioner (GP) and first specialist appointment. The times for which optimal timeframes have been recommended are the same for both lung and OG cancers.^12^, ^13^ The OCP timeframe recommendations for both lung and OG cancer patients are divided into a three‐step pathway. The first is the initial presentation to the GP for investigations and referral for a specialist appointment. Referrals to cancer specialists should not be delayed by GP investigations and these appointments should occur within 2 weeks of the GP referral. The second step in the pathways is for diagnosis, staging and treatment planning.^12^, ^13^ In Australia, cancer treatment plans are made during a multi‐disciplinary meeting (MDM) where the patient is presented by the specialist to other clinicians in diverse disciplines so that all aspects of patient care are considered before proceeding with treatment.^14^ The presentation at MDM should occur within 2 weeks of diagnosis and within 4 weeks of the GP referral, while treatment should commence within 2 weeks of the MDM. Treatment is the third and final step in the care pathway.^12^, ^13^

Improved timeliness of care has been linked to better survival among lung cancer patients.^15^, ^16^ While studies on the timeliness of access to treatment in OG cancers are scarce, one study found that a delayed surgical resection has no impact on long‐term survival.^17^ However, treatment at larger treatment centres has been shown to culminate in better survival for both lung^18^, ^19^ and OG^20^, ^21^, ^22^ cancer patients. This evidence may suggest that the larger treatment centres have more streamlined care services than smaller centres, highlighting the benefits of service centralisation.^4^ In regional areas, however, transport is a major barrier to accessing treatment. The need for regional patients to travel long distances has been associated with poor outcomes and worse quality of life.^23^ A robust, data‐driven approach to quality improvement may help to optimise services and timeliness of care in smaller, non‐metropolitan centres, with a view to overcoming the deficit relative to larger centres. Assessing the patient pathway for high‐volume tumour streams may help identify aspects of timeliness of care that are improved when centralisation is employed. We, therefore, investigated and compared timeliness of care for lung cancer, a high‐volume tumour stream, and OG cancer, a low‐volume tumour stream, at a regional health service in Victoria, Australia.

## 2 METHODS

### 2.1 Design and setting

We conducted a retrospective cohort study among patients with lung or OG cancer at a regional health service providing cancer care in the Loddon Mallee Region (LMR) of Victoria, Australia. The patient groups comprised random samples of 75 people newly diagnosed with lung cancer (International Classification of Diseases and Related Health Problems‐10 [ICD‐10] diagnosis codes C34 in the Victorian Cancer Registry [VCR]) and 50 people newly diagnosed with OG cancer (ICD‐10 diagnosis codes C15 or C16 in VCR) between 1/7/2016 and 31/12/2017 at the LMR health service. Random sampling was performed using RAND() function in Microsoft Excel (Microsoft Corp., Redmond, Washington). The region in which the study is set, the LMR makes up almost a quarter of Victoria's area yet is home to only approximately 331 000 (5% of the state's population) residents.^24^ In this region, the annual incidence of cancer is approximately 2200 cases, including 200 lung cancer cases and fewer than 100 OG cancer cases.

Using paper medical records and electronic hospital systems, data were collected on demographic variables, clinical variables and dates corresponding to optimal timeframes specified in the lung and OG cancer OCPs.^12^, ^13^ These included dates of referral receipt at the health service derived from patient letters, first specialist appointment from the clinics record, diagnosis date from the pathology results, and MDM and commencement of first treatment from the treatment logs in the patient file. Due to the exploratory nature of the study on factors associated with suboptimal timelines, all patient demographic and clinical covariates were collected. Comorbidities were categorised into the body systems affected and other cancers if present.

### 2.2 Statistical analysis

The percentage capture of each date was calculated for each tumour stream separately as well as both combined. For patients in each cancer group with relevant dates available, date pairs were used to calculate the following times in days: referral receipt to first specialist appointment, referral receipt to diagnosis, referral receipt to first treatment, MDM to first treatment, diagnosis to MDM and diagnosis to first treatment. For each time in each tumour group, the proportion of patients who did not meet the optimal timeframe published in the relevant OCP^12^, ^13^ (ie, had suboptimal timeliness of care) was calculated and expressed as a percentage. Patients who were presented at the MDM before their diagnosis were excluded from the analysis. Numbers less than 5 have been reported as “<5” to maintain patient confidentiality, in line with VCR requirements.

Patient characteristics were compared between the lung and OG cancer groups using either the *χ* ^2^ test for independence (categorical variables) or the independent samples *t*‐test (continuous variables). Continuous variables, including times with optimal timeframes specified in OCPs, were compared between lung and OG cancers using the Kruskal‐Wallis test. Factors associated with suboptimal timeliness of care (Table 3) were analysed by univariable binary logistic regression. This involved the calculation of odds ratios (ORs) and corresponding 95% confidence intervals (CIs). Multivariable binary logistic regression models, including all assessed demographic and clinical factors as comorbidities, stage of disease and Eastern Cooperative Oncology Group (ECOG) performance status,^25^ were also fit to determine if any factors were independently associated with suboptimal timeliness of care. In all analyses, missing values were excluded case‐by‐case and a *P*‐value less than .05 or a 95% CI excluding 1.00 were considered to be indicative of a statistically significant result. All statistical analyses were carried out using SPSS Version 23 (SPSS Inc., Chicago, Illinois).

## 3 RESULTS

**FIGURE 1:**
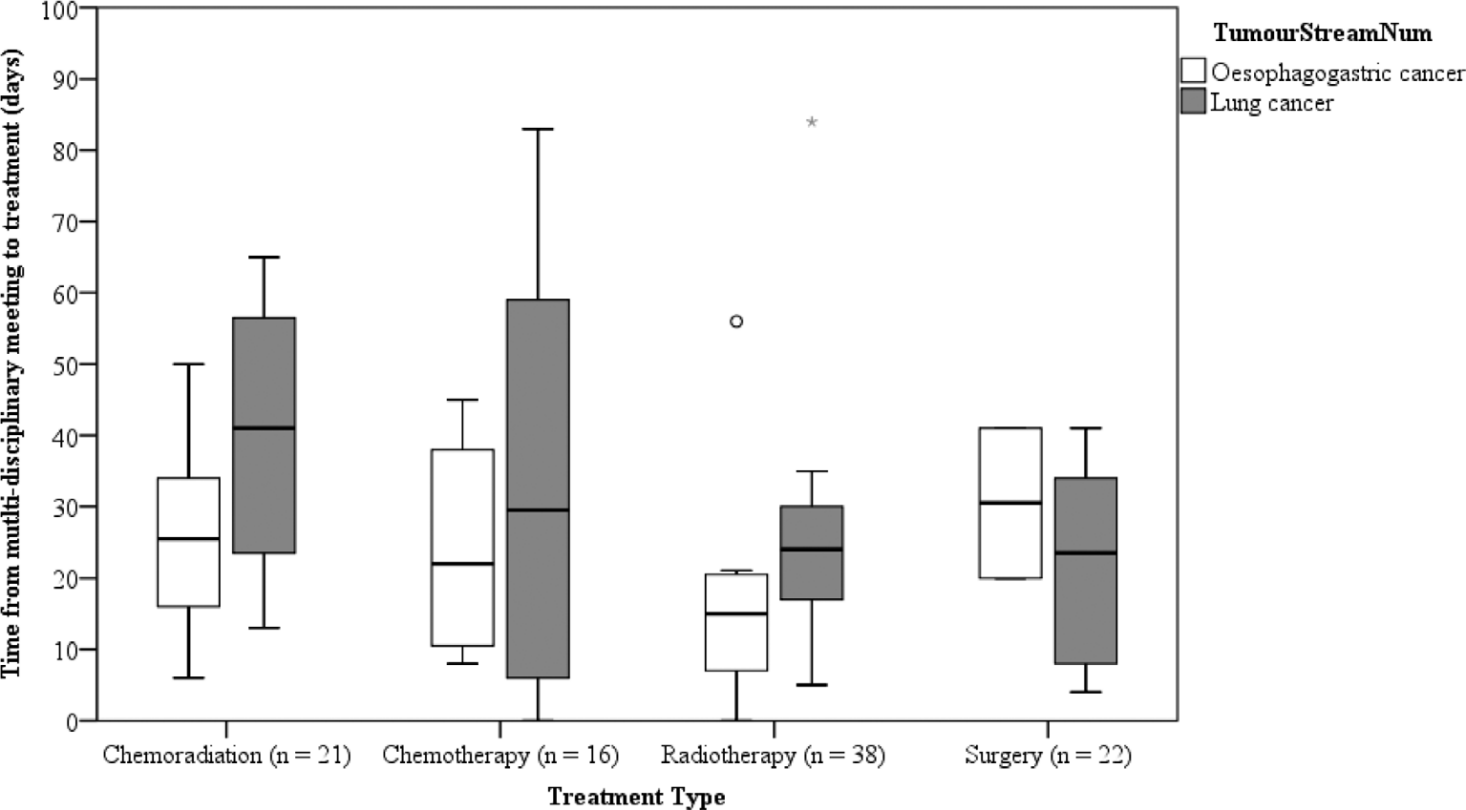
The time taken from multi‐disciplinary meeting to treatment based on the type of treatment. The box‐plots show the five‐number summary statistics: minimum, lower quartile (25th percentile), median, upper quartile (75th percentile) and maximum. (outliers are indicated by ^o^ and *)

The median ages for lung and OG cancer patients were 63.3 and 69.5 years, respectively. The older OG cancer patients were also likely to be residents of Greater Bendigo, the city in which the regional hospital is located, and had significantly more males and a significantly smaller proportion of patients with comorbidities (Table 1). The most common comorbidity were cardiovascular conditions in both lung and OG cancer patients (68% and 28%, respectively). Overall, compared with the OG cancer patient group, the lung cancer patient group had a significantly higher proportion of each comorbidity type except for comorbidities of the gastro‐intestinal tract (Table 1). Similar proportions of patients in each group had an ECOG performance status score of more than 2, a diagnosis of metastatic disease and their management options considered at an MDM (Table 1). None of the treatment types (ie, chemotherapy, chemoradiation therapy, radiotherapy of surgery) were significantly associated with suboptimal timeframes from MDM to treatment date (*P* = 0.651) when both cancer types are analysed together. However, the median times were longer for lung cancer across all treatment types except surgery (Figure 1). Due to small numbers in treatment type groups, statistical difference could not be calculated. Although the proportions of patientsvaried slightly for the different timelines, none were statistically significant (Table 2).

**TABLE 1:** Patient description in each tumour stream

Patient factor		n/N (%)^a^ Lung Cancer	n/N (%)^a^ Oesophagogastric Cancer	2‐sided *P‐val*ue^*^
Male gender (%)		42/75 (56.0%)	39/50 (78.0%)	0.009
Age in years [n (median) Q1‐Q3]		[75 (63.3)58.3‐70.3]	[50 (69.5)66.2‐73.8]	0.086
Residence in Greater Bendigo		23/75 (30.7%)	26/50 (52.0%)	0.024
Type of comorbidity	Respiratory (%)	33/75 (44.0%)	11/50 (22.0%)	0.009
	Cardiovascular (%)	51/75 (68.0%)	14/50 (28.0%)	<0.001
	Gastrointestinal (%)	20/75 (26.7%)	8/50 (16.0%)	0.118
	Metabolic (%)	40/75 (53.3%)	8/50 (16.0%)	<0.001
	Other Cancer(s) (%)	26/75 (34.7%)	<5/50 (<10.0%)	<0.001
	Bone and joint (%)	18/75 (24.0%)	<5/50 (<10.0%)	0.038
1 or more comorbidities (%)		9/75 (12.0%)	30 (60.0%)	<0.001
ECOG performance status score ≤ 2 (%)		26/32 (81.3%)	12/12 (100.0%)	0.107
Metastatic disease (%)		19/41 (46.3%)	18/29 (62.1%)	0.194
MDM (%)		58/75 (77.3%)	39/50 (78.0%)	0.555
Times between care points (days)^b^	Referral receipt to first appointment [n (median) Q1‐Q3]	[56 (12.5)5.0‐25.5]	[41 (26.5)0.75‐59.75]	0.103
	Referral to diagnosis [n (median) Q1‐Q3]	[59 (13.0)9.0‐23.0]	[32 (31.0)1.0‐75.5]	0.066
	Referral to first treatment [n (median) Q1‐Q3]	[56 (53.5)29.8‐69.5]	[34 (67.0)38.5‐112.25]	0.629
	MDM to first treatment [n (median) Q1‐Q3]	[41 (17.0)7.0‐32.0]	[29 (18.0)10.8‐31.0]	0.300
	Diagnosis to MDM [n (median) Q1‐Q3]	[36(8.0)5.0‐22.5]	[35 (19.0)11.5‐24.8]	0.353
	Diagnosis to first treatment [n (median) Q1‐Q3]	[47 (40)13.5‐60.0]	[36 (37.0)25.3‐53.5]	0.084

Abbreviations: n, number of patients with a particular factor; N, overall number of patients with data available for analysis (ie, with or without a particular factor); ECOG, Easter Cooperative Oncology Group; Q1, lower quartile (25th percentile); Q3, upper quartile (75th percentile); MDM, Multidisciplinary Meeting.

* *P*‐value from an independent samples *t* test for age and *χ* ^2^ test for independence for all other variables.

^a^ Unless otherwise specified.

^b^ Excluded cases test by test using Kruskal‐Wallis test.

**TABLE 2:** Numbers and proportions of lung and OG cancer patients with suboptimal timeliness of care (defined as timeliness outside optimal timeframes in the relevant OCPs^12^, ^13^)

Timeline	n/N (%) Lung Cancer	n/N (%) Oesophagogastric Cancer	2‐sided *P*‐value*
Referral to first specialist appointment	23/56 (41.1%)	12/41 (29.3%)	0.287
Referral to diagnosis	28/59 (47.5%)	11/32 (34.4%)	0.271
Referral to first treatment	34/56 (60.7%)	18/34 (52.9%)	0.514
Diagnosis to MDM	15/36 (41.7%)	20/35 (57.1%)	0.238
MDM to first treatment	29/41 (70.7%)	19/29 (65.5%)	0.794
Diagnosis to first treatment	21/47 (44.7%)	24/36 (66.7%)	.075

*Note*: Missing data excluded case‐by‐case.

*Note*: MDM, multidisciplinary meeting; n, number of patients with suboptimal timeliness of care; N, number of patients with each date pair available to calculate times in days.

^*^ *P*‐value from a *χ* ^2^ test for independence.

Lung and OG cancer patients were grouped together when analysing factors associated with receiving care outside optimal timeframes (ie, suboptimal timeliness of care). Table 3 shows the results of the univariable analysis for both tumour streams put together. In the univariable analysis, the odds of a suboptimal time from referral to diagnosis were 66% lower for lung cancer patients relative to OG cancer patients. However, relative to OG cancer patients, the odds of lung cancer patients having suboptimal times for diagnosis to treatment were increased 2.5‐fold. Respiratory diseases were associated with 2.4 times greater odds of a suboptimal time from referral to first specialist appointment, while other cancers were associated with 3.9 times greater odds of a suboptimal time from referral to diagnosis. The odds of a suboptimal time from referral to diagnosis were 3.8 times greater for those with metastatic disease relative to those with non‐metastatic disease. The odds of a suboptimal time from referral to first treatment were 3.2 times greater for those patients who presented with cardiovascular and gastrointestinal disorders, and 2.5 times higher in those presenting with metabolic disorders. (Table 3).

**TABLE 3:** Univariable analysis of factors associated with suboptimal timeliness of care in reference to those with optimal timeliness of care

		Univariable OR (95% CI)					
Overall Lung and Oesophagogastric cancer		Referral Receipt to First Specialist Appointment	Referral to Diagnosis	Referral to Treatment	Diagnosis to MDM	MDM to Treatment	Diagnosis to Treatment
Age at diagnosis (years)		0.99 (0.96 to 1.04)	1.03 (0.99 to 1.07)	0.98 (0.94 to 1.02)	1.02 (0.98 to 1.06)	1.01 (0.97 to 1.06)	0.99 (0.95 to 1.03)
Residence in Greater City of Bendigo		0.59 (0.24 to 1.44)	0.93 (0.40 to 2.19)	1.86 (0.77 to 4.45)	1.05 (0.41 to 2.68)	1.25 (0.44 to 3.54)	1.96 (0.81 to 4.73)
Male Gender		0.75 (0.31 to 1.79)	1.38 (0.56 to 3.37)	1.17 (0.48 to 2.86)	1.53 (0.59 to 4.05)	1.68 (0.59 to 4.82)	0.67 (0.27 to 1.69)
Lung cancer^a^		0.59 (0.25 to 1.40)	0.34 (0.14 to 0.83)*	0.73 (0.31 to 1.72)	1.87 (0.73 to 7.79)	0.79 (0.28 to 2.18)	2.48 (1.01 to 6.09)*
MDM		2.06 (0.62 to 6.88)	1.50 (0.54 to 4.17)	2.92 (0.89 to 9.56)	n/a	n/a	2.86 (0.88 to 9.27)
Comorbidity	Respiratory	2.40 (1.02 to 5.63)*	1.18 (0.50 to 2.81)	1.86 (0.77 to 4.45)	2.53 (0.92 to 6.91)	1.97 (0.68 to 5.70)	2.15 (0.86 to 5.35)
	Cardiovascular	1.17 (0.51 to 2.70)	1.50 (0.64 to 3.50)	3.16 (1.32 to 7.54)*	1.18 (0.46 to 3.00)	1.18 (0.43 to 3.25)	0.61 (0.25 to 1.47)
	Gastrointestinal	1.73 (0.68 to 4.42)	1.42 (0.51 to 3.94)	3.21 (1.06 to 9.68)*	0.75 (0.25 to 2.30)	0.52 (0.17 to 1.56)	2.17 (0.73 to 6.41)
	Metabolic	0.56 (0.24 to 1.31)	1.89 (0.78 to 4.59)	2.46 (1.01 to 5.96)*	0.82 (0.31 to 2.16)	1.40 (0.48 to 4.08)	1.04 (0.43 to 2.54)
	Cancer	1.02 (0.38 to 2.73)	3.89 (1.18 to 12.69)*	2.15 (0.79 to 5.87)	0.65 (0.17 to 2.52)	2.35 (0.60 to 9.30)	0.69 (0.23 to 2.12)
	Bone & Joint	0.78 (0.27 to 2.28)	0.85 (0.27 to 2.70)	1.43 (0.48 to 4.29)	0.62 (0.20 to 1.98)	1.34 (0.37 to 4.79)	2.40 (0.76 to 7.58)
Any comorbidity		1.06 (0.42 to 2.66)	0.49 (0.19 to 1.26)	0.41 (0.16 to 1.06)	0.73 (0.28 to 1.91)	0.48 (0.17 to 1.41)	0.87 (0.35 to 2.18)
Number of Comorbidities		0.99 (0.88 to 1.12)	0.90 (0.79 to 1.03)	0.90 (0.79 to 1.02)	0.95 (0.83 to 1.08)	0.92 (0.79 to 1.06)	0.92 (0.81 to 1.05)
ECOG ≤ 2 ^b^		0.19 (0.03 to 1.25)	5.00 (0.76 to 32.93)	0.79 (0.13 to 5.01)	0.95 (0.13 to 7.28)	9.50 (0.83 to 109.24)	0.63 (0.09 to 4.01)
Metastatic disease ^c^		1.82 (0.65 to 5.08)	3.81 (1.19 to 12.16)*	2.32 (0.76 to 7.08)	2.50 (0.74 to 8.45)	2.93 (0.78 to 10.98)	1.98 (0.66 to 5.94)
Unknown stage ^d^		0.45 (0.18 to 1.11)	0.97 (0.41 to 2.30)	0.65 (0.28 to 1.54)	1.33 (0.64 to 2.76)	0.60 (0.21 to 1.71)	0.95 (0.39 to 2.32)
Oesophagogastric cancer—Univariable							
Age at Diagnosis (years)		1.02 (0.96 to 1.07)	1.06 (<1.00 to 1.135)	1.03 (0.97 to 1.09)	1.06 (0.99 to 1.13)	1.02 (0.96 to 1.09)	0.98 (0.92 to 1.04)
Residence in Greater City of Bendigo		2.29 (0.58 to 9.03)	2.50 (0.57 to 11.01)	2.02 (0.51 to 7.94)	1.50 (0.39 to 5.81)	1.35 (0.29 to 6.38)	0.28 (0.06 to 1.31)
Male Gender		0.78 (0.16 to 3.82)	2.31 (0.38 to 14.21)	1.67 (0.31 to 8.93)	0.46 (0.08 to 2.79)	3.64 (0.50 to 26.76)	0.76 (0.12 to 4.64)
MDM		4.19 (0.46 to 37.94)	8.27 (0.88 to 78.01)	3.92 (0.37 to 42.20)	n/a	n/a	1.40 (0.20 to 9.75)
Comorbidity	Respiratory	1.92 (0.43 to 8.58)	2.00 (0.37 to 10.92)	1.50 (0.34 to 6.70)	1.71 (0.35 to 8.37)	5.25 (0.54 to 50.64)	6.60 (0.73 to 60.02)
	Cardiovascular	0.63 (0.14 to 2.88)	1.04 (0.22 to 4.91)	2.76 (0.57 to 13.29)	3.50 (0.61 to 20.13)	n/a	2.50 (0.44 to 14.23)
	Gastrointestinal	2.08 (0.38 to 11.18)	2.18 (0.31 to 15.29)	7.50 (0.79 to 71.09)	1.63 (0.26 to 10.32)	3.21 (0.32 to 32.21)	n/a
	Metabolic	n/a	0.62 (0.05 to 7.57)	1.40 (0.20 to 9.66)	1.63 (0.26 to 10.32)	n/a	2.20 (0.22 to 22.20)
	Cancer	n/a	n/a	0.88 (0.05 to 15.37)	n/a	n/a	n/a
	Bone & Joint	n/a	n/a	0.88 (0.11 to 7.05)	0.76 (0.09 to 5.81)	n/a	n/a
Any comorbidity		1.31 (0.34 to 5.09)	0.67 (0.16 to 2.82)	0.46 (0.11 to 1.85)	0.49 (0.61 to 2.45)	0.10 (0.01 to 0.95)	0.20 (0.04 to 1.11)
Number of comorbidities		1.18 (0.89 to 1.57)	0.98 (0.72 to 1.33)	0.96 (0.76 to 1.20)	0.93 (0.72 to 1.18)	0.71 (0.46 to 1.08)	0.78 (0.57 to 1.07)
ECOG ≤ 2 ^b^		n/a	n/a	n/a	n/a	n/a	n/a
Metastatic disease ^c^		2.78 (0.48 to 16.03)	1.67 (0.31 to 9.01)	1.07 (0.19 to 5.91)	0.28 (0.45 to 15.31)	2.40 (0.29 to 19.78)	3.43 (0.52 to 22.80)
Unknown stage ^d^		0.62 (0.15 to 2.51)	0.55 (0.11 to 2.73)	0.50 (0.12 to 2.06)	0.44 (0.11 to 1.85)	0.46 (0.10 to 2.22)	1.20 (0.28 to 5.15)
Lung cancer‐Univariable							
Age at Diagnosis		0.99 (0.94 to 1.04)	1.00 (0.95 to 1.06)	0.94 (0.88 to 0.99)*	0.96 (0.90 to 1.04)	1.00 (0.94 to 1.07)	0.99 (0.93 to 1.05)
Residence in Greater City of Bendigo		0.20 (0.05 to 0.82)*	0.83 (0.25 to 2.72)	2.11 (0.63 to 7.08)	0.73 (0.19 to 2.81)	1.22 (0.30 to 5.03)	6.83 (1.83 to 25.40)
Male Gender		0.85 (0.29 to 2.49)	1.54 (0.50 to 4.79)	1.12 (0.37 to 3.35)	2.29 (0.59 to 8.91)	1.42 (0.37 to 5.47)	0.40 (0.12 to 1.30)
MDM		1.19 (0.26 to 5.57)	0.44 (0.09 to 2.30)	2.81 (0.69 to 11.44)	n/a	n/a	4.22 (0.79 to 22.62)
Comorbidity	Respiratory	2.39 (0.80 to 7.12)	0.63 (0.21 to 1.91)	1.97 (0.66 to 5.91)	5.00 (1.20 to 20.92)*	1.23 (0.32 to 4.74)	2.13 (0.66 to 6.88)
	Cardiovascular	1.23 (0.38 to 4.05)	0.93 (0.27 to 3.18)	3.89 (1.15 to 13.14)*	1.00 (0.25 to 4.08)	0.17 (0.02 to 1.54)	0.46 (0.11 to 1.89)
	Gastrointestinal	1.42 (0.45 to 4.49)	0.95 (0.94 to 3.30)	2.15 (0.59 to 7.90)	0.50 (0.11 to 2.37)	0.19 (0.04 to 0.80)*	1.33 (0.36 to 4.97)
	Metabolic	0.55 (0.183 to 1.63)	1.41 (0.46 to 4.30)	3.02 (0.99 to 9.19)	0.86 (0.23 to 3.25)	0.77 (0.20 to 2.98)	1.63 (0.51 to 5.23)
	Cancer	0.93 (0.31 to 2.84)	2.74 (0.77 to 9.76)	2.37 (0.75 to 7.52)	0.59 (0.32 to 7.50)	1.83 (0.41 to 8.27)	0.70 (0.19 to 2.59)
	Bone and joint	1.10 (0.32 to 3.75)	0.63 (0.17 to 2.29)	1.62 (0.43 to 6.09)	0.73 (0.17 to 3.13)	0.64 (0.15 to 2.77)	2.59 (0.70 to 9.62)
Any comorbidity		2.33 (0.35 to 15.17)	1.84 (0.19 to 17.71)	0.19 (0.02 to 1.98)	0.23 (0.02 to 2.20)	n/a	0.81 (0.12 to 5.34)
Number of comorbidities		0.99 (0.84 to 1.16)	0.98 (0.81 to 1.18)	0.86 (0.72 to 1.03)	0.87 (0.69 to 1.06)	1.00 (0.82 to 1.22)	0.83 (0.68 to 1.02)
ECOG ≤ 2 ^b^		0.23 (0.03 to 1.59)	7.20 (1.01 to 51.39)*	0.72 (0.11 to 4.82)	0.83 (0.09 to 7.68)	6.00 (0.51 to 70.67)	0.30 (0.04 to 2.17)
Metastatic disease ^c^		1.39 (0.38 to 5.07)	7.50 (1.28 to 44.09)	4.00 (0.89 to 18.01)	2.40 (0.44 to 12.98)	3.33 (0.61 to 18.15)	1.80 (0.42 to 7.81)
Unknown stage ^d^		0.38 (0.11 to 1.26)	0.98 (0.32 to 2.99)	0.74 (0.25 to 2.20)	1.93 (0.55 to 6.73)	0.76 (0.18 to 3.25)	0.80 (0.24 to 2.66)

*Note*: NB, No significant results in the Multivariable analysis.

Abbreviations: ECOG, Eastern Cooperative Oncology Group; MDM, multidisciplinary team meeting; OR, odds ratio.

* *P* < .05.

^a^ Reference to oesophagogastric cancer.

^b^ Reference to ECOG performance score of more than 2.

^c^ Reference to localised disease.

^d^ Reference to known stage.

**FIGURE 2:**
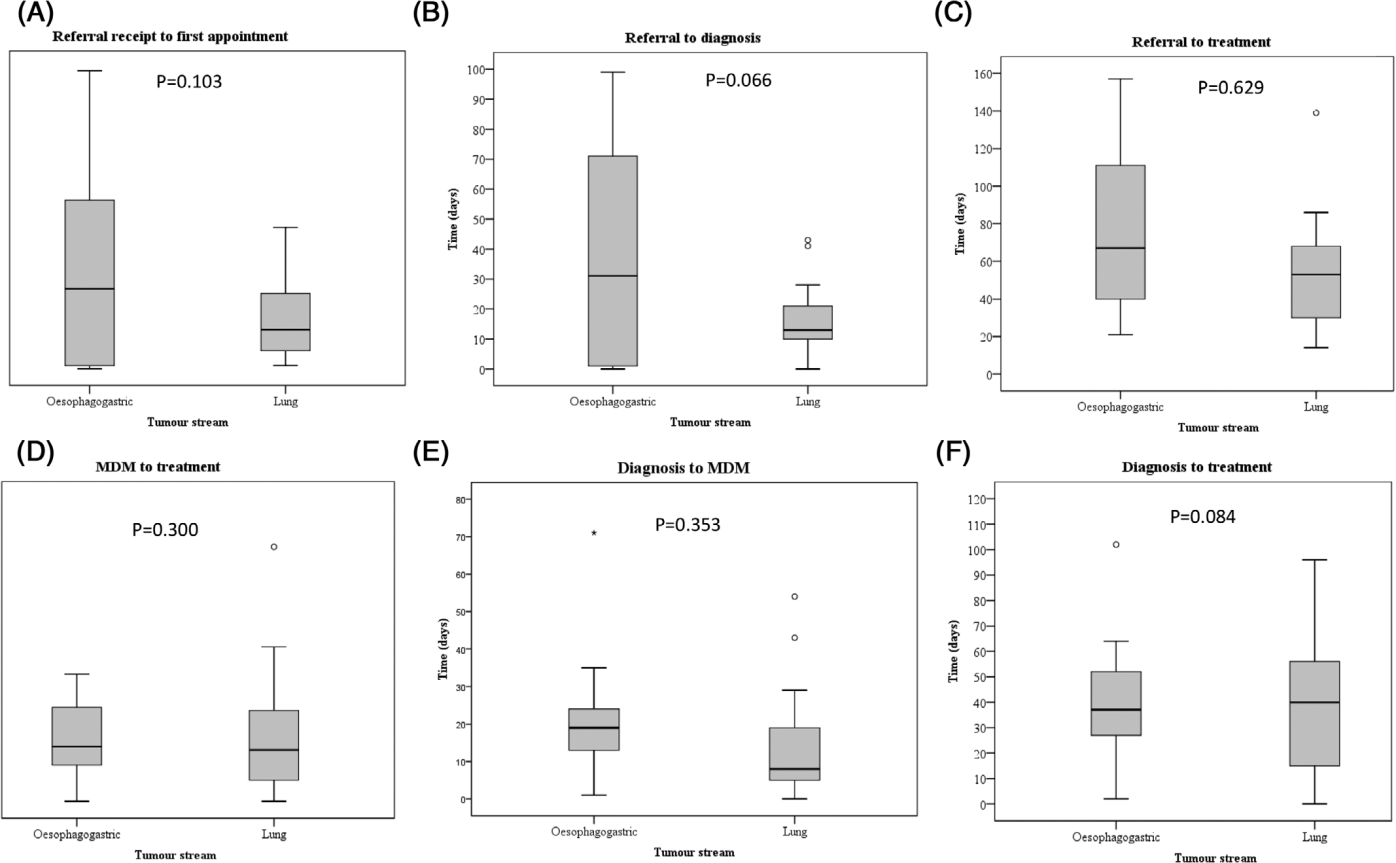
Timeframes for key points in lung and OG cancer care are shown. The box‐plots show the five‐number summary statistics: minimum, lower quartile (25th percentile), median, upper quartile (75th percentile) and maximum. (outliers are indicated by ^o^ and extreme outliers by *). Timeframes for care points shown include: A, referral receipt to first appointment (recommended within 14 days); B, Referral to diagnosis (recommended within 28 days); C, Referral to treatment (recommended within 42 days); D, MDM to treatment (recommended within 14 days); E, Diagnosis to MDM (recommended within 14 days); and F, Diagnosis to treatment (recommended within 28 days)

Factors associated with suboptimal timeliness of care were also assessed for each cancer stream individually. Among OG cancer patients, no factors were found to be associated with having suboptimal timeliness of care for any of the optimal timeframes. In lung cancer, factors associated with the odds of suboptimal care times included age at diagnosis, living within the local government area of the City of Greater Bendigo, respiratory disease, cardiovascular disease, gastrointestinal disease and presenting with an ECOG score of less than 3. The odds of suboptimal wait times from referral to first appointment were 80% lower in lung cancer patients who were residents of Greater Bendigo. However, the odds of suboptimal time from referral to diagnosis was increased in lung cancer patients presenting with metastatic disease by a factor of 7.2. The odds of suboptimal timelines from referral to first treatment were increased in lung cancer patients presenting with cardiovascular comorbidity by a factor of 3.9 and reduced 6% with an increasing age of diagnosis. The odds of suboptimal times from diagnosis to MDM were increased by a factor 5 in lung cancer patients presenting with respiratory comorbidities while the odds of suboptimal times for the time from MDM to treatment were reduced 80% when lung cancer patients who presented with a gastrointestinal tract (GIT) comorbidity. Despite all these significant univariate associations, in multivariate analyses adjusting for all other factors, no factors were independently associated with suboptimal timeframes as per OCP recommendations (results not shown). Side‐by‐side comparatives of timeliness are summarised in Figure 2 and show shorter waiting times for lung cancer patients compared to OG cancer patients for all timeliness up to diagnosis, albeit non‐significantly different except for diagnosis to MDM (Figure 2).

## 4 DISCUSSION

In this retrospective cohort study set in regional Australia, we show that the low‐volume OG cancer stream was associated with suboptimal timeliness from referral to diagnosis compared to the high‐volume lung cancer stream while the high‐volume lung cancer was associated with suboptimal timeliness from diagnosis to treatment. This may highlight the benefits of a more streamlined service as the higher volume lung cancer had better wait times for diagnostic services. The lung cancer patients had more comorbidities as well, although these together were not associated with suboptimal timeliness of care when both OG and lung cancer were analysed together. Independently, however, cardiovascular comorbidities were associated with increased odds of suboptimal wait time from referral to treatment while GIT comorbidity were associated with reduced odds of suboptimal wait time from MDM to treatment in lung cancer. Improving timeliness of care may be key to improved patient outcomes and quality of life. Compared with smaller hospitals, large‐volume hospitals have been associated with improved outcomes for both lung and OG cancer patients.^15^, ^17^ This highlights the importance of centralisation and streamlined services for improved outcomes and patient experience.

Investigating the factors associated with bringing these times within recommended timeliness becomes important in all cancers, especially low‐volume cancer types where the numbers may still be low even after centralisation. Comparing low‐volume and high‐volume cancers within one referral centre may aid in determining the influence of centralisation and hospital saturation (when hospital capacity is reached) on treatment times. Low‐volume cancer streams would give an indication of what can be done when the system is not saturated and the high‐volume on what could be improved with a saturated but streamlined service. Before undertaking this analysis, we showed that the OCPs could be mapped using paper and electronic records in both public and private hospitals in the LMR region.^26^ This study highlights some of the major differences in timeliness of care that may occur between cancers of different patient volumes. The limitations of data capture when using retrospective patient files remain, with some of the key OCP dates missing for some patients. A further potential limitation of this study is confounding of associations between covariates and suboptimal timeliness of care. As there are small numbers in the LMR, data capture and cell sizes were too low for some variables that could have otherwise been included in both univariable and multivariable analyses. While multi‐site studies may provide higher volumes of patients, they would also introduce many unknown factors that are difficult to control for in such a comparison.

Although OG and lung cancer have differences in patient volumes, they share similar five‐year survival rates^5^ and similar recommended timeliness for key aspects of care^12^, ^13^ in Australia. This regional Australian study showed similarities between cancer types in terms of patient ages, stage of disease and ECOG performance status score. The patient populations are only different in presenting comorbidities, which were more prevalent in the high‐volume lung cancer group. Although comorbidities were associated with suboptimal timeliness of care, OG cancer patients had longer wait times before treatment decisions were made. This inferred that the poorer timeliness in this population was likely related to service delays. Although the time from referral to diagnosis was shorter for lung cancer patients compared to OG cancer patients, it is important to note that the greater complexity of making a lung cancer diagnosis compared with an OG cancer diagnosis is expected to account for longer times to diagnose lung cancer. Whether the differences in timeliness are due to volume‐related efficiency in the lung cancer diagnosis process or a lack thereof in OG cancer diagnosis, remains to be investigated. Further, the differences in disease states between OG and lung cancer such as metastatic disease may have confounding effects on these findings should be interpreted with caution. Factors influencing diagnosis may contribute as well. For instance, in OG cancer, a gastroscopy going straight to the location of the problem is required. For lung cancer, there are multiple diagnostic procedures that can be and often are needed to reach a diagnosis. These may include, but are not limited to, chest X‐ray, contrast spiral computed tomography (CT), endobronchial ultrasound, CT‐guided biopsy and in rare cases, sputum cytology.^12^ For OG cancers involving a much simpler approach to diagnosis, increasing the frequency of MDMs may have potential to improve timeliness of care. Increasing MDM frequency may, however, put pressure on financial and human resources. Therefore, effective strategies for reducing costs associated with MDMs such as telehealth, may be required^27^ if such avenues are to be investigated for low‐volume cancers. At the health service where this study was conducted, the discussion of treatment plans for both OG cancer and lung cancer patients takes place in the same meeting. As there is no guideline to prioritise the presentation of one cancer type over the other, the difference in their timeliness is not a result of difficulties in organizing a meeting. For high‐volume lung cancer that was shown to have longer wait times for commencement of therapy after a diagnosis was made, options for improvement may be limited by hospital capacity.

Alleviating the effects of healthcare saturation on initiatives to improve the quality of cancer care may present its own challenges.^28^ Previously, we have shown that several sub‐regional nurse‐led oncology services with existing memorandums of understanding with the regional cancer center^29^ could be scaled up to reduce wait times and travel distances of regional Victorian cancer patients. Such services have the potential to negate the effects of hospital saturation on quality of care for high‐volume tumours.

## 5 CONCLUSION

In the low‐volume OG cancer stream, patients had longer times from referral to diagnosis but shorter time to commencement of first treatment. Conversely, in the high‐volume lung cancer group, there was delayed initiation of first treatment following presentation at MDM. There is a need to explore ways to fast track diagnoses, MDM presentations and commencement of therapy among people diagnosed with low and high‐volume cancers, respectively.

## ETHICS STATEMENT

All patient data were kept on secure drives according to the policies of Bendigo Health Care Group. All protocols were reviewed and approved by the Bendigo Health Ethics and Research Governance Committee (reference: LNR/18/BHCG/3 for the OG cancer OCP data and LNR/16/BHCG/43 for the lung cancer OCP data).

## CONFLICT OF INTEREST

Authors declare no conflict of interests.

## AUTHOR CONTRIBUTIONS

Mwila Kabwe: Formal analysis; investigation; methodology; project administration; validation; visualization; writing‐original draft; writing‐review and editing. Amanda Robinson: Data curation; software; validation; visualization; writing‐review and editing. Yachna Shethia: Data curation; investigation; software; validation; visualization; writing‐review and editing. Carol Parker: Conceptualization; validation; visualization; writing‐review and editing. Robert Blum: Supervision; validation; writing‐review and editing. Ilana Solo: Project administration; supervision; validation; writing‐review and editing. Michael Leach: Conceptualization; formal analysis; project administration; software; supervision; validation; visualization; writing‐original draft; writing‐review and editing.

## Data Availability

All data is available on reasonable request to the corresponding author
